# MicroRNAs in the Mitochondria–Telomere Axis: Novel Insights into Cancer Development and Potential Therapeutic Targets

**DOI:** 10.3390/genes16030268

**Published:** 2025-02-25

**Authors:** José Alfonso Cruz-Ramos, Emmanuel de la Mora-Jiménez, Beatriz Alejandra Llanes-Cervantes, Miguel Ángel Damián-Mejía

**Affiliations:** 1 Departamento de Clínicas Médicas, Instituto de Patología Infecciosa y Experimental, Centro Universitario de Ciencias de la Salud, Universidad de Guadalajara, Guadalajara 44340, Mexico; 2 Dirección de Desarrollo Institucional, Instituto Jalisciense de Cancerología, Zapopan 45060, Mexico; 3 Licenciatura en Nutrición, Centro Universitario de Ciencias de la Salud, Universidad de Guadalajara, Guadalajara 44340, Mexico; beatriz.llanes2764@alumnos.udg.mx; 4 Licenciatura en Médico Cirujano y Partero, Centro Universitario del Sur, Universidad de Guadalajara, Ciudad Guzmán 49000, Mexico; miguel.damian2906@alumnos.udg.mx

**Keywords:** microRNAs, telomere, mitochondria, cancer, tumor progression, carcinogenesis, *mitomiRs*

## Abstract

The mitochondria–telomere axis is recognized as an important factor in the processes of metabolism, aging and oncogenesis. *MicroRNAs* (*miRNAs*) play an essential function in this complex interaction, having an impact on aspects such as cellular homeostasis, oxidative responses and apoptosis. In recent years, *miRNAs* have been found to be crucial for telomeric stability, as well as for mitochondrial behavior, factors that influence cell proliferation and viability. Furthermore, mitochondrial *miRNAs* (*mitomiRs*) are associated with gene expression and the activity of the cGAS/STING pathway activity, linking mitochondrial *DNA* recognition to immune system responses. Hence, *miRNAs* maintain a link to mitochondrial biogenesis, metabolic changes in cancer and cellular organelles. This review focuses on the roles of a variety of *miRNAs* in cancer progression and their potential application as biomarkers or therapeutic agents.

## 1. Introduction

Mitochondria regulate the metabolism and many cellular processes, while telomeres are responsible for controlling the lifespan of the cell; impaired function in either can trigger several pathways to failure, leading to a cascade of dysfunctional events within the cell [1]. Consequently, the interaction between telomeres and mitochondria has been implicated in maintaining cellular homeostasis, contributing to the development of neoplasms and oncogenic processes, emerging as an important field of research [2].

On the other hand, it has been described that the biological behavior of a cell is mediated by the interaction of *ribonucleic acid* (*RNA*) with telomeres, specifically through telomeric repeat-containing *RNA* (*TERRA*), a non-coding *RNA* (*ncRNA*) which acquires an important function in the regulation and maintenance of telomeres [3].

This has led to research into the importance of *ncRNAs* as regulators in cell processes, emerging as a strategy for the modulation of diverse cellular pathways, such as *miRNAs*, which have demonstrated active participation in *messenger RNA (mRNA)* regulation, by binding to the 3’untranslated region (3’ UTR) [4,5]. *miRNAs* consist of small sequences of about 21 to 23 nucleotides, and their coding genes are transcribed by RNA polymerase II [5].

During malignant cellular transformation, *miRNAs* play key roles in cell differentiation by regulating cancer stem cells and tumor development [6]. Additionally, the molecular heterogeneity of cancer and its variability in the response to medical treatment may contribute to the investigation of novel therapies and diagnostic models, since even single-nucleotide polymorphisms modulate *miRNA* expression in some cancers, for example breast cancer, demonstrating a wide field of research [7,8].

Understanding the mechanisms involved in the complex axis between *miRNAs*, telomeres and mitochondria is indispensable for the development of novel approaches to cancer treatment strategies.

## 2. Cellular Organization and Energy Metabolism

Cellular organelles in eukaryotes are highly complex and maintain complex bidirectional signaling relationships with each other and with other cellular structures both functionally and structurally. The signaling pathways result in cellular metabolism regulation and evolution towards a healthy state, cell death, or malignant transformation [9]. Smaller molecules have the advantage of being able to diffuse more easily across the cell membrane compared to larger molecules. However, within the cell, specifically in the mitochondria, membranes exhibit a highly complex structure, which is closely related to their unique energy production capacity. This structural complexity not only supports energy generation but is also fundamental for cellular metabolism and the determination of cell fate, whether tending towards apoptosis, carcinogenesis, or senescence, among other cellular processes [10]. Mitochondrial membranes interact in a distinct manner with molecules and mediators, compared to the cytoplasmic membrane. In mitochondria, the membrane folds or cristae increase the surface area for adenosine triphosphate (ATP) production during electron transport in the mitochondrial respiratory chain, as well as other forms of metabolic regulation and cell proliferation [11].

Within the mitochondrial matrix resides the mitochondrial genome, which is structurally distinct from nuclear *DNA*. The mitochondrial *DNA* (*mtDNA*) in mammals is a small, circular genome of about 16.5 kb, encoding 13 subunits of the oxidative phosphorylation (OXPHOS) system, as well as the transfer *RNAs* (*tRNAs*) and ribosomal (*rRNAs*) crucial for their synthesis. The *mtDNA* system, which resembles bacterial *DNA* in its organization, is capable of synthesizing proteins that operate in close coordination with nuclear-encoded proteins imported into the mitochondria [12].

The electron transport complexes (I through V) in the inner mitochondrial membrane generate ATP mostly through OXPHOS. This process results in the formation of reactive oxygen species (ROS) as a byproduct of biochemical reactions in ATP production [13]. Additionally, mitochondria are associated with several pathways outside the tricarboxylic acid (TCA) cycle, including fatty acid oxidation and amino acid metabolism. The varying energy requirements should in principle be met by the cell through plasticity in metabolic pathways to maintain homeostasis and survive in changing local microenvironments. In this sense, the basic canonical mechanisms observed under normal conditions undergo modifications that lead to metabolic shifts in energy systems. These alterations, which are characteristic of cancer cells, include increased aerobic glycolysis and lactic acid production. Such metabolic reprogramming supports tumor progression and enhances the survival of neoplastic cells [14].

## 3. The *miRNA*: Biogenesis in Animal Cells and Its Relationship with Mitochondria

The *miRNAs* have been described in different species such as viruses, animals, and plants [15]. Nomenclature to classify *miRNAs* remains undefined; however, it has been described that *miRNAs* with identical sequences at nucleotides 2–8 of the mature *miRNA* will appertain to the same family [16].

Furthermore, to maintain a consensus for *miRNA* characterization, specific criteria are considered in addition to length; there must be a relationship with the Argonaute proteins, the synthesis must be mediated by a ribonuclease (RNase) type III, and there must be phylogenetic conservation, an abundance of sequence reads and the homogeneous presence of the 5’ end [15,16].

Several studies have elucidated these processes by studying various animal species, mainly humans, *Caenorhabditis elegans* (*C. elegans*), *Drosophila melanogaster*, and *Giardia intestinalis* [15,16]. Moreover, phylogenetic conservation among mammals has been demonstrated [16,17].

However, it is important to mention the differences between *miRNA* biogenesis in animals and plants. In plants, it has been observed that the processing takes place entirely in the nucleus and unlike in animals, and there is no homolog of Drosha and DiGeorge syndrome critical region gene 8 (DGCR8); therefore, most of the processes are mediated by DICER-LIKE 1, another type III RNase [16]. In this review, we describe *miRNA* biogenesis in animal cells, focusing on human cells.

*miRNA* biogenesis begins in the cell nucleus with the transcription of a *DNA* strand to produce primary *miRNA* (*pri-miRNA*) by RNA polymerase II [16]. This primary transcription is mediated by the complex of Drosha and DGCR8, which acts as an enzyme and removes the non-structured endings of the *pri-miRNA*, therefore creating *pre-miRNA* as a precursor [16,18]. Drosha is an RNase type III which cuts *pri-miRNA*; meanwhile, DGCR8 acts as a cofactor through which to recognize and stabilize *pri-miRNA* structure [19].

Once *pre-miRNA* is recognized by exportin 5 (XPO5), and Ras-related nuclear protein bound with Guanosine Triphosphate (RAN-GTP) provides the necessary energy, pre-*miRNA* is transported from the nucleus to the cytoplasm. Then, the RNase type III DICER, recognizes and processes the chain by cutting the hairpin; thus, a double-stranded *miRNA* of approximately 22 nucleotides is generated [16,18]. This *miRNA* duplex has two strands, the mature *miRNA*, which will be the functional guide strand, and the passenger strand, which will be degraded.

**Figure 1:**
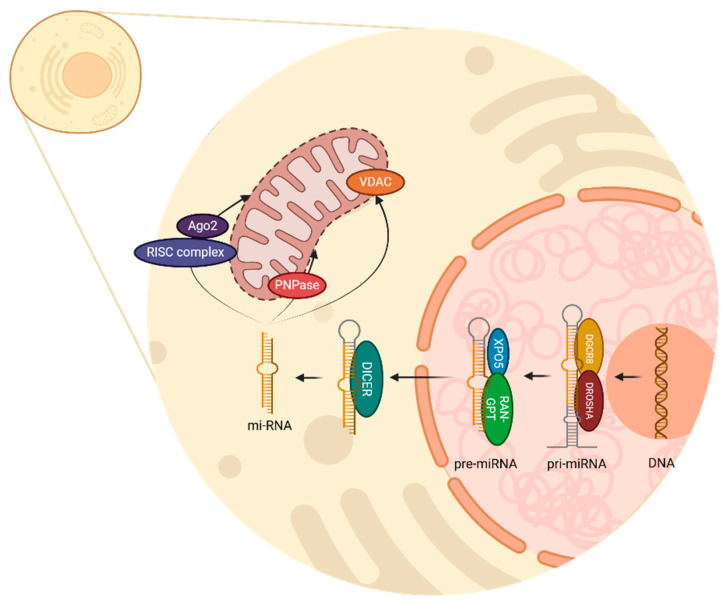
In human cells, *miRNA* biogenesis begins in the nucleus, where RNA polymerase II transcribes *DNA* into *pri-miRNA*. The DROSHA–DGCR8 complex then cleaves *pri-miRNA*, generating *pre-miRNA*, which is subsequently transported to the cytoplasm by XPO5 and RAN-GTP. Once in the cytoplasm, DICER processes the hairpin structure into a 22-nucleotide double-stranded *miRNA*, consisting of a functional mature strand and a degradable passenger strand. To modulate cellular functions, *miRNA* associates with RISC and Ago2, either promoting *mRNA* degradation or inhibiting its translation. Additionally, *miRNAs* are found within the mitochondria, where they influence gene expression, with PNPase and VDAC facilitating their transport.

In the cytoplasm, *miRNA* can conjugate with various complexes and regulate important functions. When incorporated into the RNA-induced silencing complex (RISC) and Argonaute 2 (Ago2) protein, *miRNA* can identify complementary sequences in *mRNA* and regulate two mechanisms: *mRNA* degradation and translation inhibition [20]. Additionally, it is known that several *miRNAs* can be found inside the mitochondria, accomplishing regulatory processes in mitochondrial genes. Polynucleotide phosphorylase (PNPase) facilitates the importation of *miRNA* into mitochondria, and the voltage-dependent anion channel (VDAC) participates in *RNA* and protein transportation through the external mitochondrion membrane [21,22]. All these processes are illustrated in Figure 1.

Notably, most of the *miRNAs* known are synthesized outside the mitochondrion, in other words, they are nuclear *miRNAs* with targets in the mitochondrion. Nevertheless, the difference in the process of *miRNAs* synthesized within the mitochondria compared to the process of those encoded in the nucleus remains unclear [23]. Once localized inside the mitochondrion, the presence of *miRNAs* can modulate its dynamics, which could lead to structural changes, resulting in alterations in fission or fusion, causing damage to the organism [24].

## 4. Fusion and Fission Mitochondrial Processes

Mitochondria are complex organelles that can fuse and divide to some degree, similarly to bacterial cell division. Under certain conditions, mitochondria fuse specifically depending on the context, microenvironment, and cellular stress [10,25,26]. Golgi apparatus-dependent mitochondrial fusion is achieved through GTPases such as Mitofusin 1 (MFN1) and Mitofusin 2 (MFN2), which coordinate outer mitochondrial membrane fusion. On the other hand, Optic atrophy 1 (OPA1) protein is responsible for mediating inner mitochondrial membrane fusion [9,10,25].

Dysregulation of fusion and fission promotes the survival of malignant cells; dysfunctional mitochondria lead to abnormalities in both mitochondrial morphology and function [25,26]. Fusion processes are particularly important when metabolic demand is high, and therefore mitochondria can fuse and share components to maintain their functionality and energy efficiency [10,26].

The mitochondrial fission machinery relies on dynamin-related protein 1 (DRP1) and its receptors FIS1, MFF (mitochondrial gene-specific protein), and MiD49/51 to preserve mitochondrial morphology and their distribution in the cell matrix [9,10,25]. In cancer cells, aberrant fission is observed, which further promotes the proliferation and survival of malignant neoplastic cells, making it a stress response process [9,25,26].

## 5. Mitochondrial–Telomere Communications in Cancer

### 5.1. Molecular Basis of Crosstalk Communication Between the Mitochondria and Telomeres

The bidirectional interactions between mitochondria and telomeres at the molecular level contribute in a fundamental manner to cellular homeostasis and adaptation. This interaction is more complex than previously thought, particularly regarding oxidative stress and cellular signaling pathways [27,28].

A significant breakthrough in understanding this relationship comes from studies showing that mitochondrial H_2_O_2_ release and ROS production affect nuclear *DNA* and telomeres through sophisticated signaling mechanisms rather than direct oxidative damage, challenging previous assumptions about these interactions [27,29].

The telomere–mitochondria axis is particularly relevant in cellular senescence and aging. It has been shown that telomere damage influences mitochondrial function through specific signaling pathways, notably the p53-peroxisome proliferator-activated receptor gamma 1α coactivator (PGC-1α) pathway [1]. Furthermore, Sung et al. have shown that interventions targeting this axis, such as metformin treatment, can influence both telomere stability and mitochondrial function, potentially mitigating cellular senescence [30].

This complex interplay between telomeres and mitochondria represents a critical regulatory mechanism in cellular aging and disease progression, where dysfunction in either component can trigger a cascade of cellular responses affecting both structures [1,27]. Understanding these interactions has important implications for developing targeted therapeutic strategies for age-related diseases and cancer [29,30].

### 5.2. Mitochondrial–Telomere Communication via Non-Coding RNAs

*ncRNAs* are key in mediating communication among mitochondria and telomeres. Nassour et al. research has revealed sophisticated mechanisms through which these *RNA* molecules coordinate various cellular processes, particularly in the context of senescence and cancer [31].

A key discovery involves *TERRA*, which are long non-coding *RNAs* (*lncRNAs*) transcribed from dysfunctional telomeres [32]. These *TERRA* transcripts have been shown to interact specifically with ZBP1 (Z-DNA binding protein 1) on the outer mitochondria membrane, where they form distinct oligomeric structures [33,34].

Dysfunctional telomeres trigger cellular senescence through the activation of *DNA* damage response pathways. While senescence is mediated by p53 and retinoblastoma tumor suppressor (RB) pathways, cells with disrupted checkpoints bypass this protective mechanism [35]. Therefore, these cells enter a replicative crisis characterized by transcriptional changes due to an overlap of upregulated genes [31,32]. During crisis, telomeres undergo active transcription, producing *TERRA*: *lncRNAs* sequences containing UUAGGG repeats and subtelomeric-derived *RNA* [32]. *TERRA* has been involved in the sensing innate immune system pathways through the induction of interferon-stimulated genes (ISGs) [31]. Among ISG products, ZBP1 protein has arisen as a main mediator in cell death regulation and innate immunity through the induction of type I interferons (IFNs).

The synthesis of ZBP1 seems to be linked to the cyclic GMP-AMP synthase/stimulator of interferon genes (cGAS/STING) pathway, which becomes activated upon the cytosolic accumulation of nucleic acids. Furthermore, cGAS/STING-mediated *DNA* sensing amplifies ZBP1 expression [31,36].

**Figure 2:**
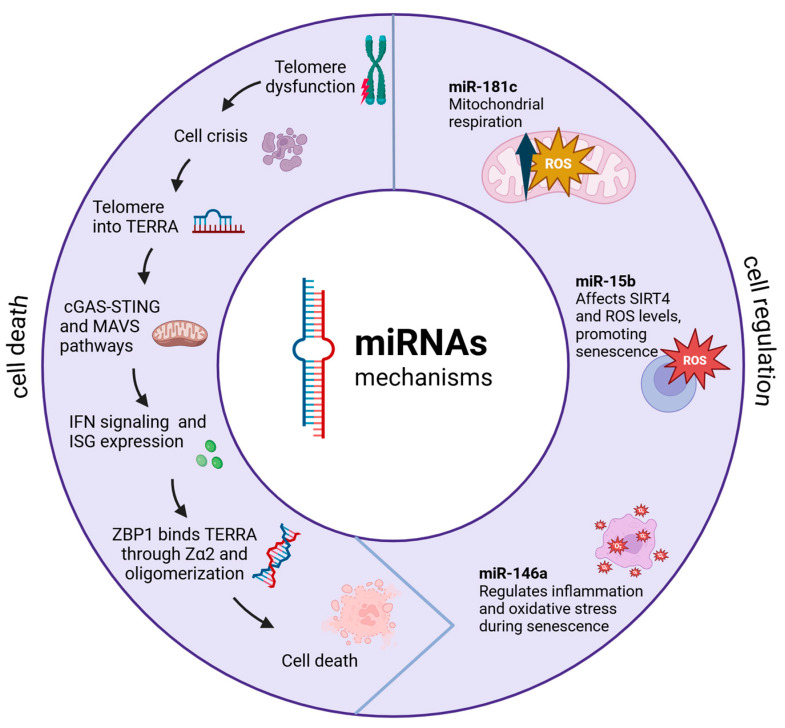
Telomere dysfunction triggers a complex molecular signaling cascade integrating innate immune responses. Telomere erosion and sheltering loss induce *TERRA RNA* transcription, which acts through two parallel pathways, including the formation of complexes with ZBP1 (Via Zα2 domain) that activate MAVS at the mitochondrial surface. These converging pathways lead to type I interferon signaling and pro-inflammatory cytokine production. The response is amplified through feedback loops involving mitochondrial ROS production and sustained IRF3/7 and NF-κB activation. This mechanism serves as a tumor suppression pathway where telomere dysfunction couples to innate immunity through mitochondrial signaling, ultimately triggering cellular senescence or cell death programs based on telomeric damage severity.

The conformation of the ZBP1–*TERRA* complex through the Zα2 domain leads to the expression of mitochondrial antiviral signaling protein (MAVS), which leads to the activation of nuclear factor kappa–B (NF-kB) and interferon regulatory factor (IRF) 3/7 signaling 170 pathways, resulting in ISG expression [31,37]. Additionally, the complete activation of IFN-dependent ZBP1 requires the previous upregulation of ZBP1 by cGAS/STING and a signal from dysfunctional telomeres. When these conditions are fulfilled, an inflammatory loop is established, leading to enhanced ISG expression and to cell death [31]. This interaction triggers a cascade of events that can lead to programmed cell death in cancer cells, representing a novel tumor-suppressive mechanism; this mechanism is represented in Figure 2.

In this sense, *miRNAs*, particularly those targeting mitochondrial functions (*mitomiRs*), have emerged as important regulators of cellular metabolism and cancer progression. These mitochondria-localized *miRNAs* can originate from both nuclear and mitochondrial genomes, impacting various aspects of energy metabolism and cellular defense mechanisms [38]. Feng et al. have shown that specific *mitomiRs* are differentially expressed in various cancer types, affecting both mitochondrial function and the metabolic reprogramming of cancer cells [39].

### 5.3. Mitochondrial Nuclear-Encoded MitomiRs

*mitomiRs* act as essential post-transcriptional regulators. Kuo et al. have shown that *mitomiRs* require specific targeting mechanisms and specialized transport systems for proper mitochondrial localization [40]. Maurya et al. have demonstrated that these regulatory molecules play crucial roles in maintaining mitochondrial function and cellular homeostasis [41].

The regulation of mitochondrial function by nuclear-encoded *mitomiRs* occurs through multiple pathways. A well-documented example is *miR-181c*, which has been shown to significantly affect mitochondrial function by modulating ROS synthesis and glucose oxidation patterns in cancer cells. Luo et al. have identified its function as a tumor suppressor and its influence on drug responsiveness, highlighting that processes such as telomere attrition, oxidative stress, activation of tumor-suppressing pathways and mitochondrial impairment serve as primary triggers of cellular senescence [20,42].

Lang et al., in recent research, reported a link between *miR-15b* and ROS, where the inhibition of *miR-15b* induces mitochondrial ROS generation and reduces the mitochondrial membrane potential-dependent upregulation of SIRT4 [43]. On the other hand, *miR-146a* has been shown to have an important role in regulation processes regarding ROS production and inflammation, by interacting with elements of the NF-kB pathway and NADPH oxidase 4 (NOX4), where it functions as an anti-inflammatory and antioxidant, resulting in a decrease in oxidative stress [44].

Disruptions in the transport, assembly, or localization of mitochondrial elements may severely affect cellular homeostasis. For example, in tongue squamous cell carcinoma, an upregulation of *miR-2392* has been demonstrated to specifically hinder *mtDNA* transcription, markedly decreasing the expression of COX1, ND4, and CYTB, ultimately inhibiting OXPHOS [20,45].

Additionally, a decline in *miR-107* levels can result in mitochondrial dysfunction, characterized by diminished mitochondrial membrane potential and impaired electron transport chain (ETC) activity. Such alterations may profoundly influence key cellular processes, including resistance to drugs, inflammatory responses, and aging [20].

Alterations in *mitomiRs* expression are closely linked to cancer metabolism and mitochondrial stress signaling. The latest evidence, according to Maurya et al., demonstrates that tumor cells process mitochondrial ROS regulation to interact with various components in the tumor microenvironment, thereby affecting cancer progression [41]. Furthermore, Dasgupta et al. investigated a novel regulatory mechanism in *mtDNA*-less cells. Their analysis revealed a reduced expression of several mitochondrial *RNAs*, including *COX1-3*, *12S rRNA*, and *ND4-5*. In contrast, *Cyto b*, *ND1* and *ND3* were entirely absent in 206 ρ° cells. This imbalance in mitochondrial *RNA* transcription indicates that post-transcriptional cleavage and processing mechanisms may be involved in controlling mitochondrial gene expression within these cells [46].

By partially base pairing with complementary sites in the cytosol, *miRNAs* are enabled to regulate mitochondrial *RNA* expression by aiming for specific *mRNAs*. This regulation of mitochondrial gene expression has significant implications for cellular energy metabolism [46,47].

### 5.4. Mitochondria-Encoded miRNAs

Mitochondrial-encoded *miRNAs* have recently begun to be studied, and while not much is known, it is understood that they are encoded in *mtDNA* and are processed by fine and specific machinery within the mitochondria [9]. These unique *miRNAs* have been shown to have distinct processing mechanisms that differ from their nuclear counterparts, suggesting specialized regulatory functions [20].

Initially, their existence was doubted; however, several studies have confirmed not only their existence but also their crucial participation in cellular metabolism and mitochondria–nucleus communication [46]. Yumeng et al. have emphasized the critical role of *miRNAs* in regulating both nuclear and mitochondrial proteins, thereby establishing a complex network of cellular regulations. Notably, abnormal methylation of mitochondrial *RNA* can impact *mtDNA* function by altering transcription stability and structure. Ribosomal *RNA* (*rRNA*) methylation is primarily regulated by mitochondrial *rRNA* methyltransferases, which include enzymes targeting both large (MRM1, MRM2, MRM3, TRMT61B) and small (TFB1M, TRMT2B, NSUN4, METTL15) ribosomal subunits [48,49].

Their processing is not traditional like nuclear *miRNAs*, suggesting a divergent evolution of these processing mechanisms. This evolutionary adaptation appears specifically aimed at regulating mitochondrial processes and facilitating communication with the nucleus [9,48]. Luo et al. have shown that these mitochondrial-specific *miRNAs* play crucial roles in maintaining cellular homeostasis and energy metabolism [20].

### 5.5. RNA-Dependent Modulation of the cGAS/STING Axis: Convergence of Mitochondrial Dynamics, Telomeric Integrity, and Programmed Cell Death Pathways

Pattern recognition receptors (PRRs) are of great importance in the innate immune system. PRRs recognize pathogen-associated molecular patterns (PAMPs) and host damage-associated molecular patterns (DAMPs) on the surface of innate immune cells [50].

The PRRs that have been mainly related to the cGAS/STING pathways are AIM2-like receptors (ALRs) and 2’-5’oligoadenylate synthetase-like receptors (OLRs) [51]. Once *DNA* is detected in the cell, ALR activates the STING-dependent ISG pathway. This mechanism was originally studied in antiviral responses [51,52]. OLRs, when activated in the cytoplasm by double-stranded nucleic acids, generate second messenger molecules like cGAMP. These molecules bind to and activate STING, triggering a downstream innate immune response [53].

cGAS activation enables the synthesis of cGAMP from GTP and ATP, which then binds to STING dimers localized in the endoplasmic reticulum (ER) membrane, resulting in their incorporation into coatomer protein complex II (COPII); then, it recruits and promotes the autophosphorylation of TANK-binding kinase 1 (TBK1), STING phosphorylation at Ser366, and the recruitment and further phosphorylation of IRF3, which is required for the induction of genes encoding IL-6 and IL-12. IRF3 dimerizes and translocates to the nucleus, ultimately leading to the expression of type I IFN and ISGs, boosting the cross-presentation of antigens to CD8+ T cells [54,55].

It was demonstrated that mitochondria experiencing minority mitochondrial outer-membrane permeability (miMOP) release *mtDNA* into the cytosol [56]. The release of *mtDNA* into the cell triggers the *mtDNA*-dependent cGAS/STING pathway, leading to the synthesis of type I IFN and inflammatory markers, which leads to the senescence-associated secretory phenotype (SASP) and inflammation [55,56].

It can be inferred that *mtDNA*-dependent cGAS/STING signaling is of great importance not only in senescence but also in certain illnesses such as cancer, where it has been shown to be key for antitumor immunity. STING activation is believed to promote tumor rejection by eliciting CD8+ T cell responses [57].

It promotes the enlistment of T cells and natural killer (NK) cells, into the malignancy microenvironment. Additionally, activation of the cGAS/STING signaling helps suppress the tumor metastasis capacity; as these cells attempt to spread, active STING signaling enhances immune recognition and destruction, thereby restricting the formation of new tumors [57,58,59].

**Figure 3:**
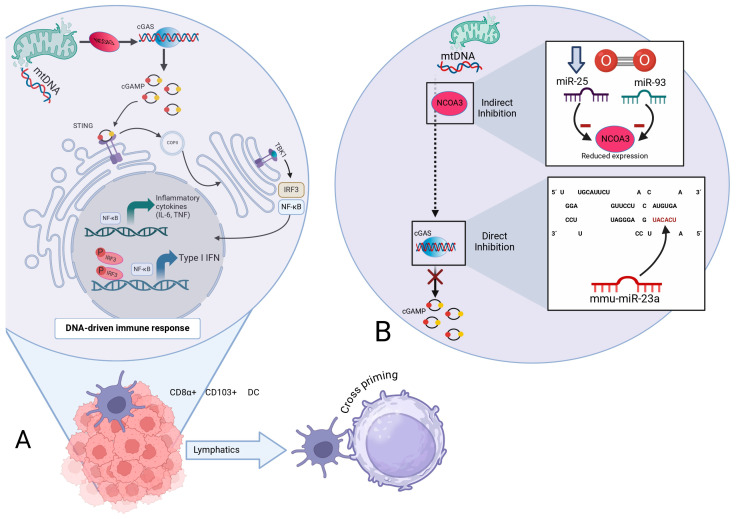
(**A**). *mtDNA*-dependent cGAS/STING signaling pathways and its suspected role in cancer. A schematic detailing how double-stranded *mtDNA* is released into the cell due to miMOP. NCOA3 aids in maintaining cGAS expression. cGAS dimers gather on *mtDNA* following the activation and synthesis of cGAMP. CGAMP then binds to stimulators of STING dimers contained at the ERr, inducing STING oligomerization and promoting COPII vesicle formation. STING then recruits TBK1, enabling its autophosphorylation: STING phosphorylation at Ser366; recruitment and phosphorylation of IRF 3 followed by its dimerization of and translocation to the nucleus, inducing the gene expression of type I IFN, as well as genes encoding inflammatory cytokines (IL-6), finally enhancing the DNA-driven immune response in dendritic cells (DCs), leading to cross priming. (**B**). Role of *miRNAs* in *mtDNA*-dependent cGAS/STING signaling. *miR-25* and *miR-93* interact with NCOA3 in the context of hypoxia, indirectly affecting its ability to positively regulate cGAS expression. On the other hand, *miR-23a/b* regulates the cGAS/STING pathway by binding directly to the 3’ region of cGAS, resulting in restrictions of the synthesis of cGAMP and therefore of Type I IFN and pro-inflammatory cytokines.

*miRNAs* play an important role in regulating the cGAS/STING pathways. The 3’UTRs of cGAS/STING *mRNA* carry probable *miRNA* binding sites [51]. Yu et al. have stated that *miRNAs* can suppress the immune response through different mechanisms. For instance, certain *miRNAs*, such as *miR-23a/b*, directly bind to the 3’ UTR of cGAS, inhibiting its expression and thereby suppressing the cGAS-mediated innate immune response [60]. Indirectly, during hypoxia, *miR-25* and *miR-93* regulate cGAS expression by targeting NCOA3, a crucial epigenetic factor for maintaining cGAS expression levels. This regulation results in the downregulation of cGAS *mRNA* levels, facilitating hypoxic tumor cells to evade immune detection by modulating the cGAS/STING pathway [61], Figure 3.

## 6. Metabolic Reprogramming in Cancer

Malignant cells are profoundly reprogrammed in terms of energy metabolism; these changes are linked to and affect telomere and mitochondrial function. Aerobic glycolysis is prominently used by tumor cells; this has been called the Warburg effect [62]. Cancer cells maintain a balance between aerobic glycolysis, which involves glucose fermentation even in the presence of oxygen, and OXPHOS, which produces ATP through the oxidation of glucose carbon bridges [63].

The balance in energy acquisition is directed towards the prolonged cellular survival characteristics of certain malignant tumor clones. This type of mixed metabolism favors the maintenance of telomere integrity, characteristic of cancer cells [64]. Thus, this type of cancer cell metabolism promotes proliferation and telomere length maintenance through the modification of the TCA cycle, amino acid metabolism, and fatty acid oxidation [64].

The AMP-activated protein kinase (AMPK)–mammalian target of rapamycin (mTOR) axis regulates cellular energy metabolism, mitochondrial function, and telomere maintenance. This pathway is tightly dysregulated in cancer cells; therefore, these changes support both metabolism and cell survival [65,66]. Also, sirtuins (SIRT), particularly SIRT1 and SIRT3, act as metabolism-associated sensors that regulate mitochondria and telomeres in cellular stress responses and cancer cell adaptation to specific contexts [67].

Central to cellular metabolism regulation is the mTOR pathway, which acts aberrantly in cancer cells and has multiple effects on cellular metabolism. Indeed, mTOR and AMPK are regarded as metabolic sensors that relay cell survival signals under stress, and such crosstalk with mitochondrial function and telomere maintenance is essential for promoting cancer [68,69]. This means that cancer cell metabolic plasticity, mitochondrial and telomere structure and function changes play an important role in the survival of neoplastic cells and their proliferation [65].

The SIRT system modulates mitochondrial and telomere physiology through interaction with multiple molecules via histone protein deacetylation and the regulation of mitochondrial function and apoptosis. These proteins regulate cell proliferation and differentiation through various catalytic activities that depend on NAD+. Additionally, due to their deacetylase activity, they interact closely with telomeres, preventing their erosion and supporting their repair. Therefore, the processes of oncogenesis and tumor progression are closely linked to these axes and metabolic pathways related to AMPK, mTOR, and SIRTs, which in turn are precisely regulated by the large family of *miRNAs*. The complexity of these systems is very high, and their effects are dependent on the cellular microenvironment. Energy metabolism is strongly linked to the regulation of these highly specific molecular systems, with pleiotropic functions in most organs and systems, both under healthy and diseased conditions [70,71].

In hepatocellular carcinoma (HCC), SIRT1 is overexpressed and correlates with tumor grade, predicting adverse clinical outcomes and poor prognosis. *miR-34a*, decreased in severe HCC cases, has been shown, in mice, to induce tumor regression and prevent recurrence when expressed. Similarly, *miRNA-29c* acts as a tumor suppressor by inhibiting SIRT1 in HCC [72,73]. However, SIRTs also have antitumoral effects or dual effects (pro- and anti-tumoral) that can be context-dependent. For example, SIRT1 is associated with protective effects against tumor growth and promotes the cell death of tumor cells. It inhibits the proliferation of various types of cancer, such as ovarian, lung, and colorectal cancer [74].

Regarding the link between *miRNA* activity and metabolic reprogramming, it is closely associated with cancer cell transformation. The *miR–200c*–SIRT2 interaction axis plays a crucial role in establishing the metabolic shift known as the Warburg effect by regulating glycolytic enzymes [75].

In summary, metabolic reprogramming in cancer is regulated by *miRNAs*, which in turn regulate the canonical metabolic pathways of malignant transformation and progression such as mTOR, AMPK, and SIRT. These pathways reversely modulate cell cycle and fate, impacting mitochondrial structure and function and telomeric maintenance, confirming that the elucidation of these processes intrinsically involves investigating cancer reprogramming or the Warburg effect, demonstrating that the mitochondria-telomere axis is fundamental in this regulation, with *miRNAs* serving as the way that all these processes are fine-tuned.

## 7. Clinical Implications and Therapeutic Applications in Cancer

The clinical applications of *miRNA* appear to cover a vast range, and yet are largely unexplored. *miRNAs* play crucial roles in various diseases. Additionally, numerous *miRNAs* have been associated in degenerative diseases and cellular senescence, including increased expression of *miR-34a*, *miR-21-22*, *miR-26b*, *miR-29* and *miR-2010*, as well as decreased expression of *miR-106a*, *miR-19b*, *miR-20a* and *miR-320c* [76].

Specifically, in the field of cancer research, *miRNAs* have garnered significant attention for their potential diagnostic, prognostic, and therapeutic applications.

The relationship between mitochondria and telomeres and their connection to senescence and malignant transformation processes represent opportunities for the diagnosis and monitoring of multiple diseases, especially oncological diseases. *RNA* species associated with oncological processes and cellular senescence can be useful as diagnostic markers and for monitoring disease progression, potentially resulting in increased specificity to traditional oncological markers [77,78].

In this sense, *miRNAs* are known to interact with several pathological conditions; high *miR-181c* expression is associated with mitochondrial dysfunction, and differences in *miR-138* levels are associated with telomere abnormalities [76]. *miRNAs*, as an entire family of transcriptional regulators and molecular receptors, are likely to be useful in clinical assessments for the identification of improved subtypes and prognostication of oncological diseases [78].

In this regard, these molecules can also be used in *RNA*-based therapy, which can be directed at specific therapeutic targets and be used as regulators of mitochondria and telomeres. This represents a new frontier in personalized medicine, as *miRNAs* can be used to regulate metabolic pathways that can be utilized according to the context of the tumor type and the pathways that are altered [76,78].

The goal is to restore cellular functions, specifically mitochondria and telomeres, through synthetic *RNA* or regulatory molecules of these *RNAs*. In the case of *miR-34a*, mimicking molecules can be developed for the treatment of certain neoplasms to regulate mitochondrial functions and telomere maintenance, thereby restoring the homeostasis of both organelles [79,80].

## 8. Current Challenges and Future Directions

Developing *miRNA*-based therapeutics is challenging and needs to address the intrinsic stability of *miRNA*-based therapeutic agents and their delivery to the cellular compartments in which they are needed to modulate cellular functions. Nanoparticle delivery and other cell-specific transport methods have been applied for reduce side effects and enhance therapeutic efficacy [81,82].

Basic and clinical research is required to understand the complex network of molecular interactions of *miRNAs* with other *RNA* species. Bioinformatics and the development of highly complex and sophisticated computational models can shed light on the network of interactions between *RNA* species, telomeres, and mitochondria, thereby enabling the development of personalized therapies [83,84].

Moreover, integrating artificial intelligence methods and machine learning can lead to precise predictions and characterizations of different types of *RNA* and their relationship with mitochondrial and telomeric function and structure [85,86].
